# Lithium decreases the effects of neuronal calcium sensor protein 1 in pedunculopontine neurons

**DOI:** 10.14814/phy2.12740

**Published:** 2016-03-31

**Authors:** Stasia D'Onofrio, Francisco J. Urbano, Erick Messias, Edgar Garcia‐Rill

**Affiliations:** ^1^ Center for Translational NeuroscienceDepartment of Neurobiology and Developmental SciencesUniversity of Arkansas for Medical SciencesLittle RockArkansas; ^2^ IFIBYNE‐CONICET‐UBAUniversity of Buenos AiresBuenos AiresArgentina; ^3^ Department of PsychiatryUniversity of Arkansas for Medical SciencesLittle RockArkansas

**Keywords:** Bipolar disorder, Gamma band activity, sleep/wake

## Abstract

Human postmortem studies reported increased expression of neuronal calcium sensor protein 1 (NCS‐1) in the brains of some bipolar disorder patients, and reduced or aberrant gamma band activity is present in the same disorder. Bipolar disorder is characterized by sleep dysregulation, suggesting a role for the reticular activating system (RAS). Lithium (Li^+^) has been shown to effectively treat the mood disturbances in bipolar disorder patients and was proposed to act by inhibiting the interaction between NCS‐1 and inositol 1,4,5‐triphosphate receptor protein (InsP_3_R). NCS‐1 is known to enhance the activity of InsP_3_R, and of Ca^2+^‐mediated gamma oscillatory activity in the pedunculopontine nucleus (PPN), part of the RAS. This study aimed to determine the nature of some of the intracellular mechanisms of Li^+^ on rat PPN cells and to identify the interaction between Li^+^ and NCS‐1. Since Li^+^ has been shown to act by inhibiting the enhancing effects of NCS‐1, we tested the hypothesis that Li^+^ would reduced the effects of overexpression of NCS‐1 and prevent the downregulation of gamma band activity. Li^+^ decreased gamma oscillation frequency and amplitude by downregulating Ca^2+^ channel activity, whereas NCS‐1 reduced the effect of Li^+^ on Ca^2+^ currents. These effects were mediated by a G‐protein overinhibition of Ca^2+^ currents. These results suggest that Li^+^ affected intracellular pathways involving the activation of voltage‐gated Ca^2+^ channels mediated by an intracellular mechanism involving voltage‐dependent activation of G proteins, thereby normalizing gamma band oscillations mediated by P/Q‐type calcium channels modulated by NCS‐1.

## Introduction

Human postmortem studies reported increased expression of high affinity, low capacity neuronal calcium sensor protein 1 (NCS‐1) in the brains of some bipolar disorder and schizophrenic patients (Koh et al. 2003). Reduced or aberrant gamma band activity has been reported in the same disorders (Ozerdem et al., 2011; Ulhass & Singer 2010). That is, gamma band activity is disrupted in the same disorders that show overexpression of NCS‐1 (Senkowski and Gallinat 2015; Wynn et al. 2015; Leicht et al. 2016). Cognitive and executive functions are associated with gamma‐band activity (Eckhorn et al. 1988; Gray and Singer 1989; Singer 1993; Philips and Takeda 2009). Additionally, gamma oscillations are essential to information processing during sensory perception, motor behavior, and memory formation (Kann et al. 2014), and are critical for communication among brain areas, thus allowing large‐scale integration of distributed sets of neurons (Rodriguez et al. 1999; Whittington et al. 2000; Nikolic et al. 2013). Large disturbances in neurocognition can be seen throughout the different stages of bipolar disorder. Symptoms of both manic and depressive episodes in bipolar disorder include sleep and circadian rhythm disturbances, emotional dysregulation, and cognitive impairment (Leboyer and Kupfer 2010). Specifically, attention and memory deficits, impairment in verbal recall and fine motor skills, and disturbance of sustained attention are present during depressive episodes, whereas during mania episodes dysfunctions are seen in attention, complex processing, memory, and emotional processing (Goldberg and Chengappa 2009). Cognitive deficits are present even during euthymia, where executive function, verbal memory, sustained attention, visual memory, and verbal fluency are disturbed (Bora et al. 2009). Considering that bipolar disorder is characterized by prominent sleep dysregulation, this indicates a role for the reticular activating system (RAS).

In a previous study examining the pedunculopontine nucleus (PPN), part of the RAS controlling waking and REM sleep, we found that NCS‐1 modulated Ca^2+^ channels in PPN neurons that generate gamma band oscillations, and that excessive levels of NCS‐1, as expected with overexpression, reduced gamma band oscillations in these cells (D'Onofrio et al. 2015). We found 1 μmol/L NCS‐1 to be the most critical concentration for gamma oscillation modulation. These findings suggest the modulation by NCS‐1 of Ca^2+^ channels presumably located on the dendrites of PPN neurons that mediate gamma band oscillations. These results also suggest that NCS‐1 overexpression may be responsible for the decrease in gamma band activity present in at least some bipolar disorder patients. Li^+^ has been shown to act by inhibiting the interaction between NCS‐1 and inositol 1,4,5‐triphosphate receptor protein (InsP_3_R) (Schlecker et al. 2006), and has also been shown to effectively treat the mood disturbances seen in bipolar disorder patients. NCS‐1 is known to enhance the activity of InsP_3_Rs (Kasri et al. 2004), thus amplifying the Ca^2+^ signal through these receptors. Importantly, InsP_3_Rs are present in the PPN (Rodrigo et al. 1993).

The goal of this study was to determine the nature of some of the intracellular mechanisms of Li^+^ within the PPN and to identify the interaction between Li^+^ and NCS‐1. Since Li^+^ has been shown to act by inhibiting NCS‐1/InsP_3_R interaction, we hypothesize that Li^+^ will reduce the effects of overexpression of NCS‐1, therefore preventing the downregulation of gamma band activity and restoring normal levels of gamma oscillations. In this study, we also found that an enhanced voltage‐dependent inhibition of Ca^2+^ channels by G‐protein‐mediated intracellular pathways underlay the effects of Li^+^ on gamma band activity in the PPN. Our findings provide a novel area of future research to determine if this intracellular mechanism is involved in the treatment of mood disturbances seen in bipolar disorder patients, and point to new therapeutic targets for alleviating some of the arousal and sleep/wake disturbances in this devastating disease.

## Material and Methods

### Slice preparation

Pups aged 9–13 days of either sex from adult timed‐pregnant Sprague–Dawley rats (280–350 g) were anesthetized with ketamine (70 mg/kg, I.M.) until tail pinch reflex was absent. This age range was selected due to the developmental decrease in REM sleep of the rat that occurs between 10 and 30 days (Jouvet‐Mounier et al. 1970). This period of investigation enabled sampling from a baseline period (9–13 days), before the epoch of the greatest transitions that peak at 14–16 days and continue until >20 days, as determined by our previous body of work on the PPN (Garcia‐Rill et al. 2008). Pups were decapitated and the brain was rapidly removed and cooled in oxygenated sucrose‐artificial cerebrospinal fluid (sucrose‐aCSF). The sucrose‐aCSF consisted of (in mmol/L): 233.7 sucrose, 26 NaHCO_3_, 3 KCl, 8 MgCl_2_, 0.5 CaCl_2_, 20 glucose, 0.4 ascorbic acid, and 2 sodium pyruvate. Sagittal sections (400 *μ*m) containing the PPN were cut and slices were allowed to equilibrate in normal aCSF at room temperature for 1 h. The aCSF was composed of (in mmol/L): 117 NaCl, 4.7 KCl, 1.2 MgCl_2_, 2.5 CaCl_2_, 1.2 NaH_2_PO_4_, 24.9 NaHCO_3_, and 11.5 glucose. Slices were recorded at 37°C while perfused (1.5 mL/min) with oxygenated (95% O_2_–5% CO_2_) aCSF in an immersion chamber for patch clamp studies as previously described (Kezunovic et al. 2011, 2012, 2013). The superfusate contained the following synaptic receptor antagonists: the selective NMDA receptor antagonist 2‐amino‐5‐phosphonovaleric acid (APV, 40 μmol/L), the competitive AMPA/kainate glutamate receptor antagonist 6‐cyano‐7‐nitroquinoxaline‐2,3‐dione (CNQX, 10 μmol/L), the glycine receptor antagonist strychnine (STR, 10 μmol/L), the specific GABA_A_ receptor antagonist gabazine (GBZ, 10 μmol/L), and the nicotinic receptor antagonist mecamylamine (MEC, 10 μmol/L), collectively referred to here as synaptic blockers (SBs). All experimental protocols were approved by the Institutional Animal Care and Use Committee of the University of Arkansas for Medical Sciences and were in agreement with the National Institutes of Health guidelines for the care and use of laboratory animals.

### Whole‐cell patch‐clamp recordings

Differential interference contrast optics was used to visualize neurons using an upright microscope (Nikon FN‐1, Nikon, USA). Whole‐cell recordings were performed using borosilicate glass capillaries pulled on a P‐97 puller (Sutter Instrument Company, Novato, CA) and filled with a high‐K^+^ intracellular solution, designed to mimic the intracellular electrolyte concentration, of (in mmol/L): 124 K‐gluconate, 10 HEPES, 10 phosphocreatine di tris, 0.2 EGTA, 4 Mg_2_ATP, 0.3 Na_2_GTP; or a high Cs^+^/QX‐314 intracellular solution (in mmol/L): 120 CsMeSO_3_, 40 HEPES, 1 EGTA, 10 TEA‐Cl, 4 Mg‐ATP, 0.4 mmol/L GTP, 10 Phosphocreatine, 2 MgCl_2_. NCS‐1 (Prospec, Ness‐Ziona, Israel) was dissolved in the intracellular solution needed to perform each set of experiments at the concentrations described in the Results, as previously described (D'Onofrio et al. 2015). All recording electrodes had 1.2 *μ*L of standard intracellular solution injected at the tip, and the remainder of the pipette was filled with the solution (18–20 *μ*L) of the concentration of NCS‐1 to be tested. This allowed control recordings to be obtained soon after patching, while also allowing NCS‐1 to diffuse into the cell. The large volume in the pipette ensured that the concentration to which the cell was exposed was stable throughout the recording period, attaining steady‐state levels of NCS‐1 for >30 min (D'Onofrio et al. 2015). Osmolarity was adjusted to ~270–290 mOsm and pH to 7.3. The pipette resistance was 2–5 MΩ. All recordings were made using a Multiclamp 700B amplifier (Molecular Devices, Sunnyvale, CA) in both current and voltage clamp mode. Digital signals were low‐pass filtered at 2 kHz, and digitized at 5 kHz using a Digidata‐1440A interface and pClamp10 software (Molecular Devices).

The recording region was located mainly in the *pars compacta* in the posterior PPN, immediately dorsal to the superior cerebellar peduncle. This area of PPN has been shown to have the highest density of cells (Wang and Morales 2009; Ye et al. 2010). Gigaseal formation and further access to the intracellular neuronal compartment was achieved in a voltage‐clamp configuration mode, setting the holding potential at −50 mV (i.e. near the average resting membrane potential of PPN neurons (D'Onofrio et al. 2015; Kezunovic et al. 2011, 2013). Within a short time after rupturing the membrane, the intracellular solution reached equilibrium with the pipette solution without significant changes in either series resistance (ranging 4–13 MΩ) or membrane capacitance values.

To study subthreshold oscillations of PPN neurons, whole‐cell patch‐clamp configuration was switched to current‐clamp mode. Average resting membrane potentials and bridge values in current clamp were 53 ± 2 mV 13 ± 2 MΩ (*n* = 33). PPN cell type I PPN neurons (LTS current), type II PPN cells (Ia current), and type III PPN neurons (LTS + Ia currents) were identified as previously described (Kezunovic et al. 2011, 2012, 2013). All these cell types manifested gamma band oscillations when membrane potential was depolarized using a 1 sec duration ramp current clamp protocol (Kezunovic et al. 2011, 2012, 2013).

### Ca^2+^ currents

Voltage‐dependent Ca^2+^ currents were studied using a high‐Cs^+^/QX314 pipette solution (in mM: CsMeSO_3_, 120; HEPES, 40; EGTA, 1; TEA‐Cl, 10; Mg‐ATP, 4; mm GTP, 0.4; phosphocreatine, 10; and MgCl_2_). Cs^+^ and TEA‐Cl are widely used K^+^ channel blockers. Ca^2+^ currents were recorded in the presence of SBs (Kezunovic et al. 2011, 2012, 2013), and the Na^+^ and K^+^ channel blockers tetrodotoxin (TTX, 3 μmol/L) and TEA‐Cl (25 μmol/L), respectively. Since PPN neurons have long dendritic arborizations that can be difficult to depolarize, we combined QX‐314 in the pipette (to block Na^+^ currents from the inside) and superfused TTX (to block Na^+^ currents from the outside) in order to prevent large unclamped voltage‐gated Na^+^ currents from being elicited during depolarization in both voltage and current‐clamp experiments. We used two different protocols to study the effects on Ca^2+^ currents. The first protocol used square voltage steps to generate PPN neuronal Ca^2+^ currents from a holding potential of −50 mV, and then depolarized up to 0 mV, in 10 mV steps. This protocol allows rapid assessment of only high‐threshold currents. By using a −50 mV holding potential, we are able to inactivate T‐type Ca^2+^ channels, centering our recordings on the high‐threshold Ca^2+^ currents (Kezunovic et al. 2011, 2013; Hyde et al. 2013). The second protocol applied three‐pulse square voltage steps (consisting of a test pulse to 0 mV, or prepulse, for 20 msec followed by a strong depolarizing step to +50 mV for 20 msec, a brief return to holding potential of −50 mV for 10 msec, followed by another test pulse to 0 mV, or postpulse for 20 msec. Pulse durations were designed to allow full recovery of high threshold, voltage‐dependent Ca^2+^ currents) in order to study G‐protein modulation of PPN calcium currents by Li^+^. Both series resistance and liquid junction potential were compensated (>14 kHz correction bandwidth; equivalent to <10 *μ*sec lag). No significant rundown due to intracellular dialysis of PPN neuron supra‐ or subthreshold activity was observed during our recording period (up to 50 min). Fast compensation was used to maintain the series resistance <9 MΩ.

### Drug application

Bath applied drugs such as SBs were administered to the slice via a peristaltic pump (Cole‐Parmer, Vernon Hills, IL), and a three‐way valve system such that solutions reached the slice 1.5 min after the start of application. The Na^+^ channel blocker TTX, and the SBs were purchased from Sigma Aldrich (St. Louis, MO). Cholinergic antagonists were purchased from Sigma Aldrich, mecamylamine (MEC, a nicotinic receptor antagonist), as well as tetraethylammonium (TEA, a wide‐range K^+^ channel blocker). NCS‐1 (human recombinant) was purchased from Prospec Protein Specialist (Ness‐Ziona, Israel). The effects of NCS‐1 on single‐cell oscillatory activity were studied by allowing passive diffusion of NCS‐1 (with 1.2 *μ*L of standard intracellular solution first loaded into the pipette tip, followed by 18–20 *μ*L of the concentration of NCS‐1 to be tested) intracellularly through the recording micropipette, during extracellular superfusion of synaptic blockers, channel blockers, and TTX in aCSF extracellular solution. Channel blockers were purchased from Alomone laboratories (Jerusalem, Israel). We used *ω*‐Agatoxin‐IVA (*ω*‐AgA; 100 nmol/L), a specific P/Q‐type channel blocker, and *ω*‐Conotoxin‐GVIA (*ω*‐CgTx; 2.5 μmol/L), a specific N‐type channel blocker.

### Data analysis

Off‐line analyses were performed using Clampfit software (Molecular Devices, Sunnyvale, CA). As stated above, we used 1 sec duration ramps applied every 5 min in current clamp in the presence of SBs and TTX to record membrane oscillations in all three PPN cell types. Peak oscillatory amplitude was analyzed by first filtering each ramp recording and measuring the three highest amplitude oscillations to derive a mean amplitude induced during each ramp. The mean peak frequency of the same three oscillations was filtered (high pass 10 Hz, low pass 120 Hz) and measured to derive a mean frequency of oscillations during the three highest amplitude oscillations in each ramp. The power of each frequency was also analyzed by composing a power spectrum for the frequencies in the entire ramp, giving a measure of peak power for frequency. Comparisons between groups were carried out using one‐way ANOVA, with Bonferroni post hoc testing for multiple comparisons. For comparisons of effects of each concentration of Li^+^ over time, we used repeated measures ANOVA with Bonferroni post hoc testing using SAS Proc Mixed software (SAS Institute, Inc, Cary, NC). F values and degrees of freedom are reported for all linear regression ANOVAs. Differences were considered significant at values of *P* ≤ 0.05. All results are presented as mean ± SE.

## Results

Whole‐cell patch clamp recordings were performed in a total of 85 single PPN neurons to assess the effect of Li^+^ on intrinsic membrane properties, as well as to determine whether Li^+^ would affect the action of NCS‐1. The neurons were localized in the *pars compacta* in the posterior PPN, which is easily identified in sagittal sections of the brainstem (Simon et al. 2010; Kezunovic et al. 2011). We first identified PPN neurons by cell type as previously described (Garcia‐Rill et al. 2007, 2008; Simon et al. 2010). No difference in average resting membrane potential was observed among PPN neuronal types. We previously showed that, regardless of cell type, voltage‐dependent, high‐threshold N‐ and P/Q‐type calcium channel activation mediates beta/gamma frequency oscillatory activity in all PPN neurons (Kezunovic et al. 2011). We studied intrinsic membrane oscillations in 33 PPN neurons using 1 sec long depolarizing current ramps, in the presence of SBs and TTX. Depolarizing 1 sec current ramps were used to determine the voltage dependence of their oscillatory behavior as previously described (Kezunovic et al. 2011, 2013). To test the effects of Li^+^ in PPN neurons, we first bath applied Li^+^ alone at three different concentrations of 0.1, 1 and 10 mmol/L (*n* = 20). We previously reported that NCS‐1 at 1 μmol/L concentration decreased the amplitude of these oscillations (D'Onofrio et al. 2015). A group of neurons were patched to study the effects of NCS‐1 at 1 μmol/L concentration (*n* = 5). We used Li^+^ (1 mmol/L) and NCS‐1 (1 μmol/L), to study the effects of Li^+^ on ramp‐induced gamma frequency oscillations in the presence of NCS‐1 (*n* = 8). We then tested 52 PPN neurons to study Ca^2+^ currents: 29 neurons were tested in the presence of Li^+^ only and 23 were tested using both Li^+^ and NCS‐1. In order to identify N‐ and/or P/Q‐type Ca^2+^ channels, we added either *ω*‐CgTx (*n* = 35) to block N‐type channels, or *ω*‐Aga (*n* = 17) to block P/Q‐type channels, during Ca^2+^ current recordings.

### Effects of Li^+^ on the oscillatory activity of PPN neurons

We wanted to determine the effects of Li^+^ and how different concentrations affect the frequency and amplitude of Ca^2+^ channel‐mediated oscillations in single PPN neurons. Since our previous findings showed that PPN neurons cannot be effectively depolarized beyond −25 mV using square steps due to the activation of K^+^ channels during rapid depolarization (Kezunovic et al. 2011, 2013), we studied the effects of Li^+^ using a 1 sec depolarizing ramp, gradually changing the membrane potential from resting values up to 0 mV in current‐clamp mode, to induce membrane oscillations in all three groups of cells present in the PPN. The protocol applied a 1 sec duration current ramp that reached a maximum of 700 pA, executed shortly after breaking into the cell and every 5 min thereafter, for up to 30 min. We tested concentrations of Li^+^ at 0.1 mmol/L, 1 mmol/L, and 10 mmol/L (*n* = 20). Li^+^ reached maximum effect when it was bath applied for 15 min after rupturing the membrane and gaining stable access to the intracellular compartment of PPN cells.

#### Oscillation amplitude

**Figure 1:**
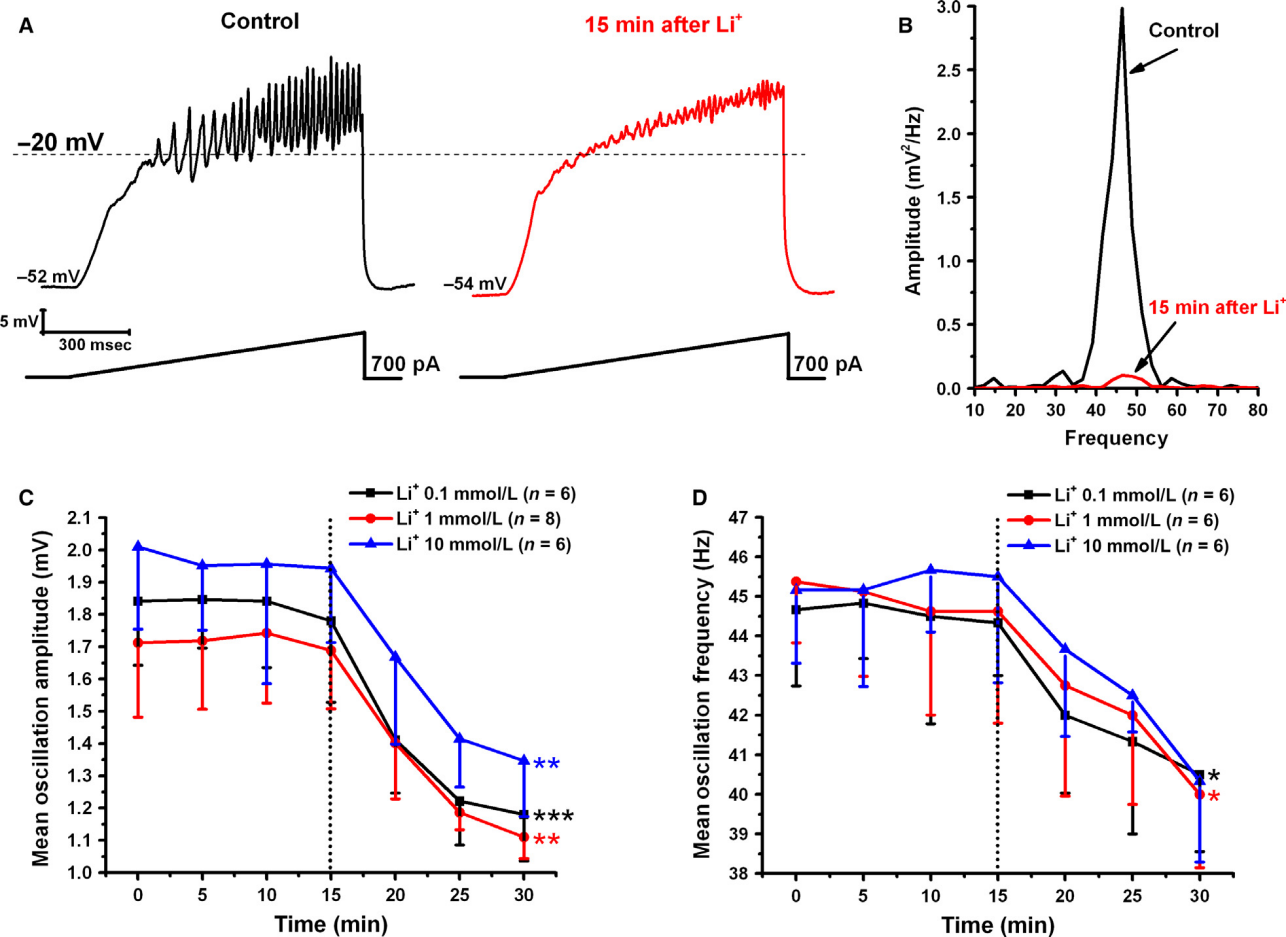
Effects of Li^+^ on ramp‐induced oscillations in PPN neurons. (A) Representative 1 sec long current ramp‐induced oscillations in a PPN neuron in the presence of SB + TTX extracellular solution (left record, black). After superfusion with 1 mmol/L Li^+^ for 15 min, oscillation amplitude and frequency were reduced (right record, red). (B) Power spectrum showing amplitude and frequency from the records shown in (A). Adding Li^+^ to the extracellular solution decreased amplitude and frequency of oscillations after 15 min exposure (red record). (C) The graph shows the mean peak amplitude (mV) of oscillations calculated by measuring the three highest amplitude oscillations after filtering to derive mean amplitude. Li^+^ was added to the extracellular solution at the 15 min time point (black dotted line). Cells recorded using 0.1 mmol/L Li^+^ showed significant decreases in amplitude 15 min after bath applied Li^+^ (black). Cells recorded using 0.1 mmol/L (black), 1 mmol/L (red), and 10 mmol/L Li^+^ (blue) showed a significant decrease at 30 min. *P < 0.05. (D) This graph shows the mean peak frequency calculated from the same three oscillations used to measure peak amplitude. Li^+^ was added to the extracellular solution at the 15 min time point (black dotted line). Cells recorded using 0.1 mmol/L Li^+^ showed significant decrease in frequency at the 30 min time point or 15 min after bath applied Li^+^ (black record). Cells recorded using 1 mmol/L Li^+^ showed significant decrease in frequency 15 min after bath applied Li^+^ (red record), but cells recorded using 10 mmol/L Li^+^ (blue record) showed no significant changes in mean peak oscillation frequency. *P < 0.05.

Mean peak amplitude was measured by taking the mean of the three peak amplitude oscillations in each ramp after filtering. During membrane potential recording in PPN neurons (in the presence of SBs and TTX), all concentrations of Li^+^ significantly decreased oscillation amplitude ~15 min after bath application. Figure 1A is an example of a representative ramp‐induced membrane potential oscillation observed in a PPN neuron (left record, black) using a 1 sec ramp protocol. After 15 min of exposure to Li^+^, oscillations were significantly reduced (right record, red). Figure 1B is a power spectrum of the records seen in Figure 1A, which shows the control recording (black line) as well as the recording 15 min after Li^+^ (red line). Similar results were obtained using all Li^+^ concentrations tested. Figure 1C shows the mean peak amplitude of ramp‐induced oscillations in cells recorded using 0.1 mmol/L (black squares), 1 mmol/L (red circles), and 10 mmol/L (blue triangles) Li^+^ concentrations. When using 0.1 mmol/L bath applied Li^+^, the mean oscillation amplitude at the beginning of the recording (0 min) was 1.8 ± 0.5 mV, which gradually decreased after application of Li^+^ (dotted line) until it was significantly reduced to 1.1 ± 0.3 mV at 30 min (df = 6, *F* = 5.29, *P* < 0.001). Mean oscillation amplitude with 1 mmol/L Li^+^ at the beginning of the recording (0 min) was 1.7 ± 0.2 mV, which decreased significantly to 1.1 ± 0.1 mV 15 min after application of Li^+^ at the 30 min time point (df = 6, *F* = 3.79, *P* < 0.01). When using 10 mmol/L Li^+^, the mean oscillation amplitude at the beginning of the recording (0 min) was 2.0 ± 0.3 mV, which decreased significantly to 1.3 ± 0.2 mV 15 min after Li^+^ application (df = 6, *F* = 4.68, *P* < 0.01). No significant differences were found comparing the maximum effect across the Li^+^ concentrations used (df = 2, *F* = 0.94, *P* > 0.05). These results suggest the modulation of Ca^2+^ channels by Li^+^ in the whole 0.1–10 mmol/L concentration range and a reduction in gamma band oscillation amplitude.

#### Oscillation frequency

Mean peak frequency was analyzed as the mean frequency calculated for the three peak amplitude oscillations in each ramp. Figure 1D shows the mean peak frequencies of ramp‐induced oscillations in cells recorded using Li^+^ 0.1 mmol/L (black squares), 1 mmol/L (red circles), and 10 mmol/L (blue triangles). The mean oscillation frequency at the beginning of the recording (5 min) was 45 ± 1.5 Hz, which gradually decreased after application of 0.1 mmol/L Li^+^ (black squares) until it was significantly reduced to 41 ± 2 Hz at 30 min (df = 5, *F* = 7.10, *P* < 0.05). At 1 mmol/L Li^+^ concentration (red circles), mean oscillation frequency at the beginning of the recording (0 min) was 45 ± 1.5 Hz, which decreased significantly to 40 ± 2 Hz after application of Li^+^ at 30 min (df = 7, *F* = 6.57, *P* < 0.05). There were no significant changes in mean peak frequency when using 10 mmol/L Li^+^ (blue triangles). It was evident that 1 mmol/L Li^+^ was an effective concentration that was used throughout the rest of the experiments.

### Effects of Li^+^ on NCS‐1 in PPN neurons

**Figure 2:**
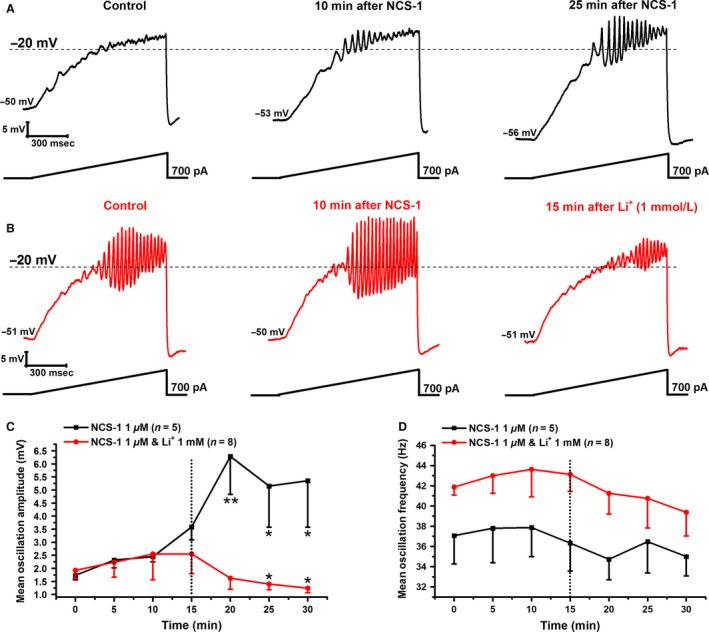
Effects of Li^+^ and NCS‐1 on ramp‐induced oscillations in PPN neurons. (A) Representative 1 sec long current ramp‐induced oscillations in a PPN neuron in SB + TTX extracellular solution, and 1 *μ*m NCS‐1 in the recording pipette (left record, black). After 10 min of NCS‐1 diffusing into the cell, the oscillatory activity increased slightly (middle record, black). However, after 25 min of NCS‐1 diffusion oscillation amplitude was significantly increased (right record, black). (B) Representative 1 sec long current ramp‐induced oscillations in a PPN neuron in SB + TTX extracellular solution and 1 *μ*m NCS‐1 in the recording pipette (left record, red). After 10 min of NCS‐1 diffusing intracellularly, the amplitude of oscillations increased slightly (middle record, red). However, after 15 min of 1 mmol/L Li^+^ in the extracellular solution, oscillation amplitude was significantly reduced below the initial recording (right record, red). (C) This graph shows the mean peak amplitude in mV of oscillations calculated by measuring the three highest amplitude oscillations after filtering to derive mean amplitude. Li^+^ was added to the extracellular solution at the 15 min time point (black dotted line). Cells recorded using only 1 *μ*m NCS‐1 (black squares), showed significant increases in mean peak oscillation amplitude at 20 min and remained increased throughout recording. Cells recorded using 1 *μ*m NCS‐1 followed by bath applied Li^+^ at 15 min showed an increase in mean peak oscillation amplitude up to 15 min, followed by a significant decrease in amplitude at both 25 min and 30 min (red circles). *P < 0.05. (D) This graph shows the mean peak frequency calculated from the same three oscillations used to measure peak amplitude. Li^+^ was added to the extracellular solution at the 15 min time point (black dotted line). Cells recorded using 1 *μ*m NCS‐1 did not show changes over time in mean peak frequency (black squares). Similarly, cells recorded using 1 *μ*m NCS‐1 followed by bath applied Li^+^ at 15 min showed no significant changes over time (red circles). However, there was a gradual numerical decrease in mean peak frequency after Li^+^ application up to 30 min.

In our previous studies, we found that NCS‐1 at 1 μmol/L promoted gamma band oscillations in PPN neurons by increasing both amplitude and peak power at all frequencies tested, while higher concentrations (10 μmol/L) appeared to block these effects (D'Onofrio et al. 2015). As expected, during membrane oscillation recordings in PPN neurons (in the presence of SBs and TTX), 1 μmol/L NCS‐1 increased amplitude of ramp‐induced oscillations. Figure 2A is a representative ramp‐induced membrane potential recording in a PPN neuron in the presence of SBs and TTX (left record, black). After 10 min (middle record, black), NCS‐1 began to numerically increase the amplitude of oscillations. However, after 25 min of recording, NCS‐1 at 1 μmol/L significantly increased the amplitude of oscillations (right record, black). Mean oscillation amplitude using 1 μmol/L NCS‐1 at the beginning of recording (0 min) was 1.7 ± 0.2 mV, increased significantly to 6.3 ± 1.5 mV after 20 min, and remained near this level (df = 6, *F* = 4.12, *P* < 0.001 for ANOVA; post hoc at 20 min *P* < 0.002; 25 min *P* < 0.04; and 30 min *P* < 0.03). Mean oscillation frequency with 1 μmol/L NCS‐1 was 37 ± 3 Hz at min 0, which did not change significantly during the ramps applied at 5 min through 30 min (df = 6, *F* = 0.30, *P *= NS for ANOVA). The effects of NCS‐1 suggest that the modulation of Ca^2+^ channels activation of PPN neurons promoted gamma band oscillation amplitude, but not frequency.

We then tested the effects of Li^+^ at the intermediate, effective concentration (1 mmol/L) tested previously in the presence of NCS‐1. After allowing NCS‐1 (1 μmol/L) to diffuse into the neuron, we bath applied Li^+^ (1 mmol/L) at 15 min. Figure 2B shows an example of ramp‐induced membrane oscillations recorded shortly after patching (left record, red). After 10 min of NCS‐1 diffusing into the cell, the amplitude and frequency of the oscillations were increased slightly (middle record, red). However, adding Li^+^ (1 mmol/L) to the extracellular solution antagonized the effects of NCS‐1 on oscillatory activity of the same PPN neuron after 15 min (right record, red). Figure 2C is a graph showing the mean peak amplitude of oscillations in neurons recorded with NCS‐1 only (black line), and neurons in the presence of both NCS‐1 and Li^+^ (red line). As described above, NCS‐1 significantly increased oscillation amplitude at 20 min and thereafter. This result suggests a gradual tripling effect on oscillation amplitude as NCS‐1 diffused into the cell. However, after 10 min of bath applied Li^+^ **,** oscillations were significantly reduced in the presence of NCS‐1 (red line). Mean oscillation amplitude with 1 μmol/L NCS‐1 at the beginning of recording (0 min) was 1.9 ± 0.3 mV, which gradually increased to 2.6 ± 0.7 mV after 15 min, followed by a significant decrease to 1.4 ± 0.2 mV at the 25 min time point (10 min after Li^+^), and decreased further at the 30 min time point, 15 min after Li^+^ exposure to 1.2 ± 0.2 mV (df = 6, *F* = 3.00, *P* < 0.03 for ANOVA; post hoc for 25 min *P* < 0.05; and for 30 min *P* < 0.03). Figure 2D shows mean oscillation frequency in the same group of cells. Mean oscillation frequency with 1 μmol/L NCS‐1 in the pipette was 42 ± 1 Hz at min 0, which did not change significantly through 30 min (df = 6, *F* = 0.70, *P* > 0.05) or after 15 min of Li^+^ (df = 6, *F* = 0.64, *P* > 0.05). These results suggest that Li^+^ has the ability to reverse the enhancing effect of NCS‐1, thereby decreasing Ca^2+^ channel‐mediated oscillations below initial levels.

### Effects of Li^+^ on Ca^2+^ currents in the PPN mediated by a voltage‐independent G‐Protein mechanism

**Figure 3:**
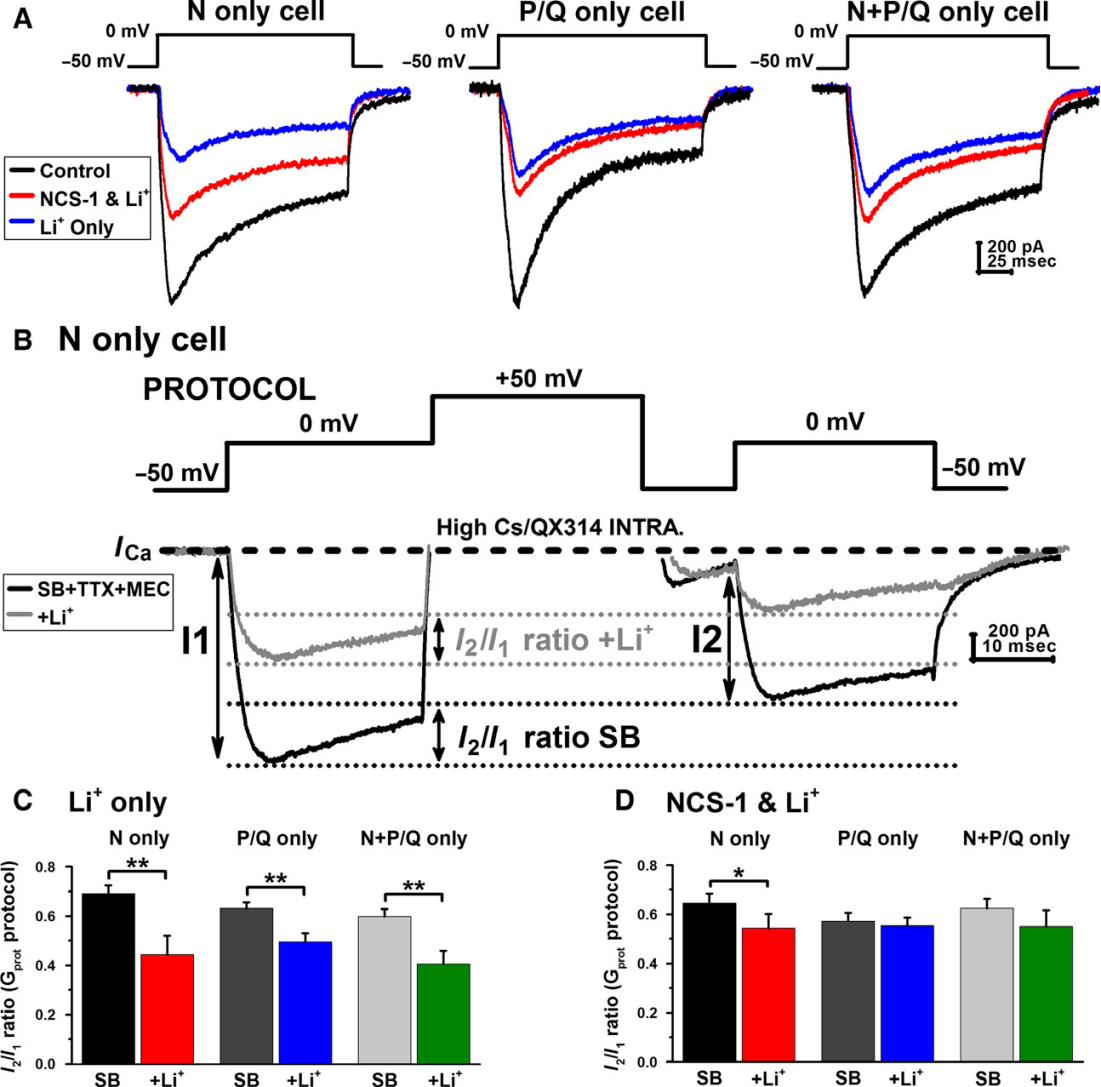
Effects of Li^+^ and NCS‐1 on voltage‐gated Ca^2+^ currents and voltage‐dependent G‐protein modulation in PPN cells. (A) Using a −50 mV depolarizing step, Ca^2+^ currents were recorded in the presence of SB + TTX + TEA‐Cl from cells expressing N only, P/Q only and N + P/Q channels. Ca^2+^ currents recorded from a cell expressing N only channels (left panel, black record) showed a reduction after application of Li^+^ (blue record). The effects of Li+ were reduced in the presence of NCS‐1 (1 μmol/L) (red record). Ca^2+^ currents recorded from a cell with only P/Q‐type channels (middle panel, black record) were also reduced after the application of Li^+^ (blue record). However, NCS‐1 had less of an effect on restoring Ca^2+^ currents in P/Q only cells (red record). Ca^2+^ currents recorded from a cell with N‐ and P/Q‐type channels (right panel, black record) showed a reduction after Li^+^ (blue record). Again, the effects of Li^+^ were prevented in the presence of NCS‐1 (red record). (B) Three‐step protocol (see Material and Methods) used to study the voltage dependence of G‐protein modulation of calcium currents (*I*_C__a_) in PPN neurons. In the presence of SBs and TTX,*I*_C__a_ (black record) were reduced in amplitude when the three‐step protocol was applied. The *I*_C__a_ seen after the second 0 mV pulse (*I*_2_) was lower in amplitude than the one seen after the first pulse (*I*_1_), yielding a *I*_2_/*I*_1_ ratio <1 in all neurons recorded. Interestingly, Li^+^ (1 mmol/L) reduced the total amount of current during both pulses while also enhancing the voltage‐independent inhibition of Ca^2+^ channels through G‐protein modulation in N‐type cells (gray record). Li^+^ had the greatest effect on *I*_2_/*I*_1_ ratio in cells expressing N‐type channels (black dotted lines), reducing the Ca^2+^ current amplitude in *I*_2_ *I*_1_ ratios by almost 50% (gray dotted lines). (C) Li^+^ (1 mmol/L) significantly reduced Ca^2+^ current amplitude in *I*_2_/*I*_1_ ratios in cells expressing N‐type channels (red column), P/Q‐type channels (blue column), and N‐ and P/Q‐type channels (green column). (D) The effects of Li^+^ were reduced in recordings with NCS‐1 (1 μmol/L) in the pipette in cells expressing N‐type channels (red column), P/Q‐type channels (blue column), and N‐ and P/Q‐type channels (green column). However, the interaction between Li^+^ and NCS‐1 was most prominent in recordings of cells expressing only N‐type channels. These results suggest that when these cells were intracellularly filled with NCS‐1 (1 μmol/L), N‐type calcium channels were more likely blocked by NCS‐1, resulting in a smaller population of channels to be further blocked by Li^+^.

We examined the effects of Li^+^ on high‐threshold voltage‐dependent Ca^2+^ currents (*I*_Ca_) present in PPN neurons (*n* = 52). Voltage‐clamp recordings were obtained using square voltage steps in combination with high Cs^+^/QX314 intracellular pipette solution in the presence of SBs, TTX, and TEA‐Cl (see Material and Methods). Ca^2+^ currents were recorded shortly after gaining access to the neuronal intracellular compartment and the series resistance was compensated and stable. The holding potential was initially clamped at −50 mV and then depolarized up to 0 mV, in 10 mV square steps. These square voltage steps were applied every 5 min for up to 30 min. Recordings of the Ca^2+^ currents lasted for up to 30 min without significant rundown, as previously reported (Kezunovic et al. 2011, 2013). Figure 3A shows the results of Ca^2+^ currents recorded during a −50 mV depolarizing step, as well as the protocol used. In order to determine the proportion of Ca^2+^ channel cell types, the specific calcium channel blockers *ω*‐CgTx (2.5 μmol/L) or *ω*‐Aga (100 nmol/L) (both individually) were added to the extracellular solution 15 min after initial recording. Cell types N only, P/Q only, or N + P/Q (Luster et al. 2015) were classified using toxins that were continuously superfused for 15 min, allowing a maximal effect to be achieved based on previous findings (Kezunovic et al. 2012). When recording from N only cells, Li^+^ (1 mmol/L) reduced total *I*_Ca_ peak amplitude by 62 ± 5% (left panel, blue record) when compared with the control condition (left panel, black record). A lesser effect by Li^+^ was seen in P/Q only cells (middle panel), which reduced total *I*_Ca_ peak amplitude by 52 ± 7% in these PPN neurons (middle panel, blue record). Similar results were seen in cells expressing both N‐ and P/Q‐type channels (right panel), where Li^+^ reduced total *I*_Ca_ peak amplitude by 49 ± 5% (right panel, blue record). We examined the effect of intracellular NCS‐1 (1 μmol/L) on the *I*_Ca_ reduction caused by Li^+^. The effect of Li^+^ was reduced in all three channel types (N only, P/Q only and N + P/Q cells) after adding NCS‐1 intracellularly (see Material and Methods; Fig. 3A, red record), however, the total blocking effect of Li^+^ was significantly reduced in N‐type cells (*n* = 15, df = 14, *F* = 5.793, *P* < 0.05 for ANOVA).

Next, we studied the voltage‐independent modulation of Ca^2+^ channels by G‐protein‐mediated intracellular pathways underlying the effects of Li^+^ alone (1 mmol/L) and NCS‐1 + Li^+^ (1 μmol/L) on gamma band activity in the PPN. Numerous studies have shown that membrane‐delimited inhibition of voltage‐dependent Ca^2+^ channels is affected by *βγ* subunits of neurotransmitter activated G proteins (Bean 1989; Kuo and Bean 1993; Herlitze et al. 1996; Ikeda 1996; Kammermeier et al. 2000). In order to study G‐protein‐mediated modulation of Ca^2+^ currents, we used a three‐step protocol that has been previously used in other models (Fig. 3B, PROTOCOL) (Avissar et al. 1988; Beck et al. 2013). This allowed us to measure Ca^2+^ currents before and after depolarization‐induced displacement of G‐protein binding. Using the appropriate conditions to record only the effects of Ca^2+^ currents (Kezunovic et al. 2011, 2012, 2013), the protocol consists of a test pulse to 0 mV (prepulse, to test the effects on Ca^2+^ currents when G proteins are bound) followed by a strong depolarizing step to +50 mV (which partially displaces G‐protein binding), a brief return to the holding potential of −50 mV, and lastly, another test pulse to 0 mV (postpulse, which tests the effects on Ca^2+^ currents when G proteins are partially unbound) (Avissar et al. 1988; Beck et al. 2013). When any given agent, such as NCS‐1 and/or Li^+^, reduces the amplitude of the prepulse more than the postpulse, a voltage‐independent G‐protein mechanism underlies such reduction in Ca^2+^ currents. On the other hand, when a similar inhibition is observed at both test pulses, then a voltage‐dependent mechanism can be described. In the presence of SBs, TTX, and TEA‐Cl extracellularly and Cs^+^/QX‐314 intracellularly, the total *I*_Ca_ seen after the postpulse (*I*_2_) was always of lower amplitude than the one observed after the prepulse (*I*_1_), yielding an *I*_2_/*I*_1_ ratio <1 in every PPN neuron studied (Fig. 3B, black record). Li^+^ (1 mmol/L) reduced the total amplitude elicited by both pulses (Fig. 3B, gray record) and was observed to have the greatest effect on *I*_2_/*I*_1_ ratio in cells expressing N‐type channels (black dotted lines), reducing the Ca^2+^ current amplitude *I*_2_/*I*_1_ ratio by almost 50% when compared to control condition (gray dotted lines). Figure 3C shows the results of the G‐protein *I*_2_/*I*_1_ ratio using Li^+^ alone (left panel) and NCS‐1 + Li^+^ (right panel). Bath applied Li^+^ significantly reduced Ca^2+^ current amplitude in *I*_2_/*I*_1_ ratios in N only cells (red column; *n* = 8, Paired *t*‐test, *t* = 3.787, df = 6, *P* < 0.01), P/Q only cells (blue column; *n* = 9, Paired *t*‐test, *t* = 4.546, df = 8, *P* < 0.01), and in N + P/Q cells (green column; *n* = 13, Paired *t*‐test, *t* = 4.944, df = 12, *P* < 0.001), when compared with the control condition (black column; dark gray column; light gray column). The effect of Li^+^ was reduced (right panel) in the presence of intracellular NCS‐1 (1 μmol/L). NCS‐1 prevented the reduction of *I*_2_/*I*_1_ ratios in N only cells (red column; *n* = 8), P/Q only cells (blue column; *n* = 6), and N + P/Q cells (green column; *n* = 9), however, NCS‐1 significantly reduced the effects of Li^+^ in recordings specifically on N only PPN neurons (Paired *t*‐test, *t* = 3.351, df = 7, *P* < 0.05). These results suggest that when these cells were intracellularly filled with NCS‐1 (1 μmol/L), N‐type Ca^2+^ channels were more likely blocked by NCS‐1 resulting in a smaller population of channels to be further blocked by Li^+^.

## Discussion

Briefly, these results show that, (1) Li^+^ caused a decrease in the amplitude and frequency of Ca^2+^ channel‐mediated, high‐frequency oscillations in PPN neurons; (2) Li^+^ significantly reduced the enhancing effect of NCS‐1 on these oscillations; (3) Li^+^ significantly downregulated Ca^2+^ channel activity regardless of channel type; while (4) the presence of NCS‐1 reduced the effect of Li^+^ on Ca^2+^ currents; and (5) an intracellular mechanism involving voltage‐independent activation of G proteins mediated these effects.

Regardless of concentration used, Li^+^ reduced the amplitude and frequency of gamma band oscillations in PPN neurons. These results suggest that, even at the very low concentrations tested, Li^+^ reduced these oscillations (Fig. 1). From a clinical standpoint, then, use of this agent would tend, in a normal individual manifesting high‐frequency oscillations during the alerted states of waking and REM sleep, to reduce arousal or REM sleep drive. Considering this, a common symptom present in bipolar disorder is insomnia (Berger et al. 2003), which is an overactive waking system intruding into sleep time (Garcia‐Rill 2015). Insomnia seen in bipolar disorder could be a result of increased levels of NCS‐1 that lead to increased high‐frequency activity and ultimately, hyperarousal of the waking system. Li^+^ may be able to alleviate the hyperarousal by downregulating the interaction between NCS‐1 and InsP3, thus decreasing high‐frequency activity. This theory is supported by evidence that Li^+^ treatment has been seen to increase slow wave sleep and reduce REM sleep (Friston et al. 1989; Zamboni et al. 1999; Qureshi and Lee‐Chiong 2004; Jones et al. 2008; Ota et al. 2013), as well as increase REM sleep latency (Campbell et al. 1989). The downregulation by Li^+^ of NCS‐1 interactions with InsP3 reduces the abnormal high‐frequency activity and restores proper rhythms to these systems.

Interestingly, the potentially fatal Li^+^ toxicity effects on cardiac function could be a result of the interaction between Li^+^, NCS‐1, and InsP3. Notably, NCS‐1 is mainly expressed in the brain and heart (Weiss and Burgoyne 2001; Nakamura et al. 2003). NCS‐1 has been identified as a novel regulator of cardiac Ca^2+^ signaling. Furthermore, a study done on immature hearts found that NCS‐1 physically and functionally interacts with InsP3. Stimulation of InsP3 resulted in phosphorylation of CaMKII‐*δ*, which was enhanced by NCS‐1 overexpression. These results indicate that a functional link exists between NCS‐1, InsP3 function, and CaMKII activation that potentially affect global Ca^2+^ signals (Nakamura et al. 2011). Li^+^ intoxication can result in cardiac conduction disturbances (Delva & Hawken 2001), and heart disease increases the risk of developing Li^+^ intoxication (Numata et al. 1999; Tielens et al. 1999). It is possible that too much Li^+^ disrupts normal Ca^2+^ signaling and activity, thus causing physiologic and electrochemical changes in the heart leading to abnormal rhythms. Therefore, we speculate that Li^+^ at optimal therapeutic levels will restore proper rhythmicity by inhibiting the effects of NCS‐1/InsP3 pathways, however, too much will result dysfunctional activity.

In addition, Li^+^ blunted NCS‐1‐mediated enhancement of high‐frequency oscillations (Fig. 2) such as those previously described in PPN neurons (D'Onofrio et al. 2015). This suggests that, in individuals overexpressing NCS‐1, Li^+^ would downregulate NCS‐1 and perhaps restore gamma band oscillations. Although others have described how therapeutic levels of Li^+^ can inhibit the intracellular interaction between NCS‐1 and InsP_3_R (Schlecker et al. 2006), our study identifies one mechanism of Li^+^ action as a key regulator of neuronal high‐frequency rhythmicity. Indeed, both gamma band oscillations and total *I*_Ca_ steady‐state reductions were only counteracted by increasing free intracellular NCS‐1, suggesting some overlapping effects.

Both P/Q and N‐type Ca^2+^ channel modulation by G proteins can involve either voltage‐dependent or independent mechanisms (Kuo and Bean 1993; Herlitze et al. 1996; Kammermeier et al. 2000). In PPN neurons, M2 muscarinic receptors mediated a voltage‐dependent inhibition of Ca^2+^ currents (Kezunovic et al., 2011; Beck et al. 2013). However, the present results showed a G‐protein‐mediated voltage‐independent inhibition was mediated by Li^+^ that further reduced *I*_2_/*I*_1_ *I*_Ca_ ratio values. Such an effect on G‐protein‐dependent Ca^2+^ channel modulation may be mediated by a Li^+^ blockade of G*α* subunits GTPase activity (Avissar et al. 1988). Voltage‐independent G‐protein inhibition of P/Q‐type channels has been found to require NCS‐1 (without altering channel opening kinetics [Weiss and Burgoyne 2001]), suggesting how increasing NCS‐1 intracellular concentration was able to prevent the full magnitude of Li^+^ effects on “P/Q only” and “N + P/Q” cells, but only partially in “N only” PPN cells. Finally, *I*_2_/*I*_1_ ratio values were not significantly altered by NCS‐1, suggesting that Li^+^/NCS‐1 intracellular pathways only affected the basal activity of Ca^2+^ channels, as previously described for inward rectifying channels (Farhy Tselnicker et al. 2015).

In summary, Li^+^ treatment would compensate the effects of overexpression of NCS‐1 (Koh et al. 2003) and of the reduced gamma (Ozerdem et al. 2011) observed in some bipolar disorder patients, perhaps by partially preventing the action of excessive NCS‐1 and restoring intracellular pathways mediating normal gamma band activity.

## Conflict of Interest

None declared.
